# VAI-B: a multicenter platform for the external validation of artificial intelligence algorithms in breast imaging

**DOI:** 10.1117/1.JMI.10.6.061404

**Published:** 2023-03-20

**Authors:** Fernando Cossío, Haiko Schurz, Mathias Engström, Carl Barck-Holst, Apostolia Tsirikoglou, Claes Lundström, Håkan Gustafsson, Kevin Smith, Sophia Zackrisson, Fredrik Strand

**Affiliations:** a Karolinska Institute, Department of Oncology-Pathology, Stockholm, Sweden; b Karolinska University Hospital, Department of Radiology, Stockholm, Sweden; c Collective Minds Radiology, Stockholm, Sweden; d West Code Group, Stockholm, Sweden; e Linköping University, Center for Medical Image Science and Visualization (CMIV), Linköping, Sweden; f Linköping University, Department of Medical Radiation Physics, Department of Health, Medicine and Caring Sciences, Linköping, Sweden; g Royal Institute of Technology (KTH), Division of Computational Science and Technology, Stockholm, Sweden; h Lund University, Department of Diagnostic Radiology, Translational Medicine, Malmö, Sweden; i Skåne University Hospital, Department of Imaging and Physiology, Malmö, Sweden

**Keywords:** breast cancer, data management, machine learning, validation, mammography

## Abstract

**Purpose:**

Multiple vendors are currently offering artificial intelligence (AI) computer-aided systems for triage detection, diagnosis, and risk prediction of breast cancer based on screening mammography. There is an imminent need to establish validation platforms that enable fair and transparent testing of these systems against external data.

**Approach:**

We developed validation of artificial intelligence for breast imaging (VAI-B), a platform for independent validation of AI algorithms in breast imaging. The platform is a hybrid solution, with one part implemented in the cloud and another in an on-premises environment at Karolinska Institute. Cloud services provide the flexibility of scaling the computing power during inference time, while secure on-premises clinical data storage preserves their privacy. A MongoDB database and a python package were developed to store and manage the data on-premises. VAI-B requires four data components: radiological images, AI inferences, radiologist assessments, and cancer outcomes.

**Results:**

To pilot test VAI-B, we defined a case-control population based on 8080 patients diagnosed with breast cancer and 36,339 healthy women based on the Swedish national quality registry for breast cancer. Images and radiological assessments from more than 100,000 mammography examinations were extracted from hospitals in three regions of Sweden. The images were processed by AI systems from three vendors in a virtual private cloud to produce abnormality scores related to signs of cancer in the images. A total of 105,706 examinations have been processed and stored in the database.

**Conclusions:**

We have created a platform that will allow downstream evaluation of AI systems for breast cancer detection, which enables faster development cycles for participating vendors and safer AI adoption for participating hospitals. The platform was designed to be scalable and ready to be expanded should a new vendor want to evaluate their system or should a new hospital wish to obtain an evaluation of different AI systems on their images.

## 1 Introduction

Machine learning (ML) is rapidly progressing in many scientific fields, including medicine.^1^ For breast cancer, we are beholding the emergence of commercially available artificial intelligence (AI) systems for breast cancer detection (AI-CADe),^2^^–^^8^ triaging, diagnosis, and risk assessment.^9^ If the performance of such systems proves to be accurate and robust in a clinical setting, incorporating them into the screening process can significantly benefit both the hospital and the screening participants. The most common use case, AI-CADe, would reduce radiologist workload and potentially reduce the rate of missed cancers.^10^ Some challenges remain despite the potential impact of adopting these technologies in the screening process. Collectively these challenges are known as the AI translational gap, which is currently under special investigation by the Federal Drug Administration in the United States of America (USA).^11^

Independent validation of the performance of such AI-CADe systems is crucial, as they have the potential to affect the well-being of breast cancer screening participants severely. Evaluating such systems requires a test set, which in the context of mammography-based breast cancer detection, should be an independent and diverse dataset that captures as much patient and equipment variability as possible. This independent test set should consist of radiological images that have not been part of the training process of any of the tested ML algorithms. This way, the algorithms’ generalizability can be best evaluated. In addition, for a specific hospital considering procuring an AI-CADe algorithm, they would be able to explore how each algorithm performs on their images. Obtaining these data is relevant for the hospital since the performance of the algorithm may vary with population characteristics and mammography equipment.

There is an imminent need to establish an independent validation platform addressing the requirements described above. However, its implementation holds several challenges, mainly due to data privacy and the computational resources required. Vendors of commercial AI-CADe systems often provide their products as a web service to self-manage the required infrastructure (usually consisting of resource-intensive and specialized hardware). Sending images to their web services implies that the vendors obtain access to sensitive medical information. The privacy of individuals is protected by regulatory frameworks such as the General Data Protection Regulation (GDPR) in the European Union; therefore, information-sharing with AI-CADe system vendors through web services is challenging. Moreover, sharing an anonymized or pseudonymized (term defined in Sec. 4) version of the test set with such companies carries the risk that images meant for testing are used for training AI-CADe systems. One way to overcome the data transfer challenges is to evaluate the systems locally at the hospital. However, most hospitals do not have the infrastructure to evaluate these systems in-house. Therefore, an external validation platform can provide a solution where the AI-CADe vendors do not have access to the test data, the hospitals do not have access to the vendors’ intellectual property, and each hospital would not need to invest in the necessary computational and human resources.

In this paper, we present the multicenter validation of artificial intelligence for breast imaging (VAI-B) platform, currently with data from three hospitals being processed by three AI-CADe systems for mammography-based breast cancer detection. We describe the process of extracting the data, obtaining the inference results from the AI-CADe systems, and loading a database with all the information needed to analyze their performance while preserving data privacy and security. It addresses the challenge of external validation of AI-CADx algorithms in mammography by describing a hybrid platform that combines cloud-based and on-premises solutions for privacy and scalability. Another strength is that our platform, as opposed to challenge-oriented platforms, is intended to serve as a permanent resource that developers of AI-CADx algorithms can continuously access to prove the validity of their algorithms and determine if future algorithm versions constitute improvements or not. A third strength is that, we provide a NoSQL database schema and the accompanying code for data storage and organization, which is released as a readily available open-source python package. The paper is structured as follows: first, we introduce the organizational context of the effort; then, we describe the data components that are managed by the platform and how they are extracted from the sources; then, we demonstrate the implementation of the platform in the cloud and on-premises; and finally, we discuss the data privacy and safety considerations.

## 2 Organizational Context

Finding an appropriate organizational format for an independent multicenter validation platform is challenging as many requirements exist. A first concern is proximity to regular healthcare operations to ensure that the analytical results are of clinical relevance and to lower the perceived threshold of using the platform for clinical decision-makers. To this end, the governance model of VAI-B was designed to reside with the relevant National Program Areas of medical diagnostics and cancer care, which consists of established professional committees promoting the development of healthcare practices in Sweden. Since this governance would be functional also in future upscaled operations, a key sustainability component is in place from the start. Another requirement is trustworthiness, which is needed among care providers and AI system vendors. In addition to national governance, the credibility of the platform builds on the academic merits of its scientific leadership (primarily authors FS and SZ) and of its home organization, Karolinska Institute. Credibility also stems from SZ and FS leading the national professional organization for breast radiology (The Swedish Society of Breast Radiology). Another credibility contribution is that VAI-B is connected to an incubator for validation platforms within the national community analytic imaging diagnostics arena (AIDA), with substantial experience sharing clinical imaging data.^12^

## 3 Data

**Fig. 1:**
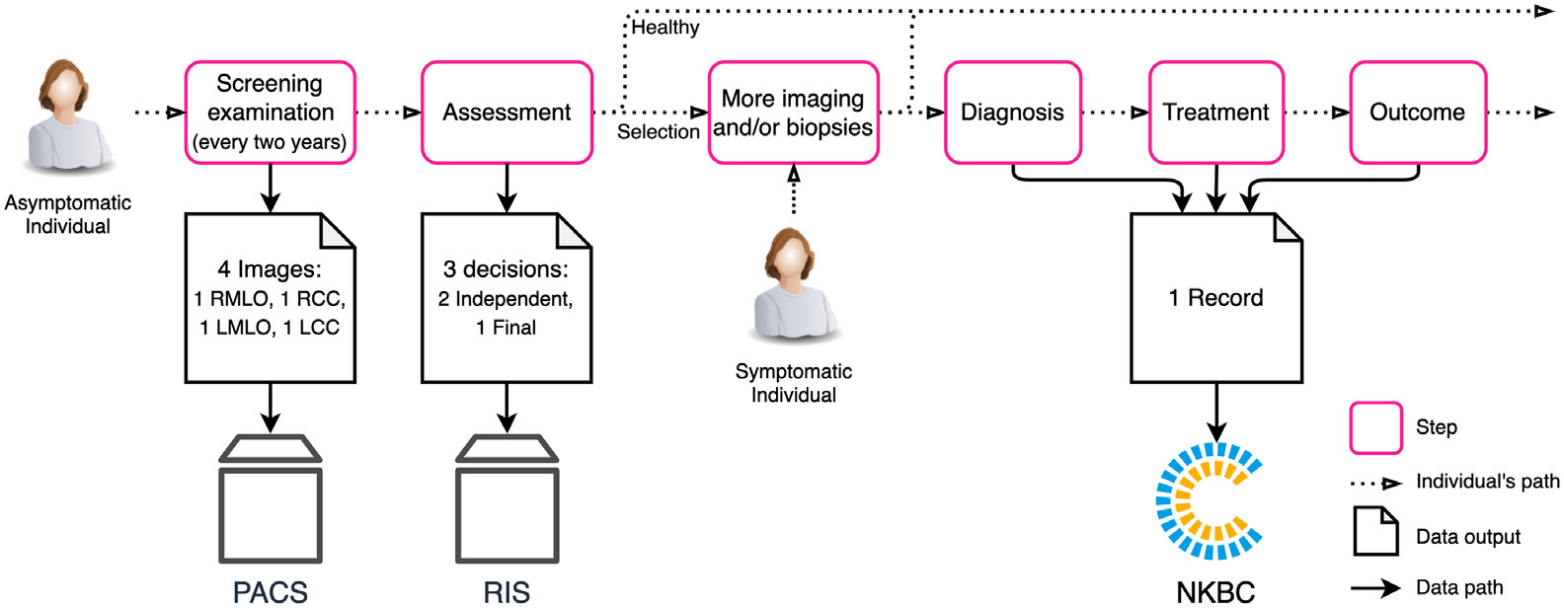
Simplification of the individual’s journey through Sweden’s current breast cancer screening program. The diagram shows where the relevant data is stored (Images in PACS, assessments in RIS, and cancer outcomes in NKBC). The PACS and RIS systems are controlled by each participating hospital, while the NKBC is a national-level organization. For the asymptomatic individual that goes through the screening examination (also regarded as a participant), there are records of the assessments done by two radiologists. For all individuals that get diagnosed, there exists a record in NKBC.

There are four data components required for the independent validation of an AI system: radiological images (“images” for brevity), AI system inferences (the inferred likelihood that an image presents signs of cancer) (“inferences” for brevity), human radiologist assessments (“assessments” for brevity), and cancer outcomes. The AI system processes the images to create inferences, which can be compared against the cancer outcomes and the assessments. As individuals get examined, the radiological data is acquired at the screening facilities and stored in a picture archiving computer system (PACS) and a radiology information system (RIS). In Sweden, the cancer outcomes data is reported from the hospitals to the Swedish National Breast Cancer Quality Register (NKBC), as shown in Fig. 1.

For the extraction from the hospitals (images and assessments), the principal investigator (author FS) contacted the legal data owner at each radiology department. Along with the request for data, the hospital received a copy of the ethical approval and a description of the data handling, including measures to ensure GDPR compliance. After review, written approval was obtained from each hospital to extract the requested data. For NKBC, the standardized data extraction procedure, defined by the organization as a request form, was sent by the principal investigator to the Regional Cancer Center Stockholm–Gotland, which manages the NKBC data. They forwarded it to the legal data owner of the registry, who decided to allow the requested data extraction.

### 3.1 Images

The Digital Imaging and Communications in Medicine (DICOM) standard from the National Electrical Manufacturers Association (NEMA) simplifies real-world concepts and activities of medical imaging into the following information model^13^: A “patient,” in the DICOM standard, is the individual that is examined; a “study” is an ordered procedure where one or multiple modalities can be used to create images of a patient, and it is commonly defined by the combination of a unique patient and calendar day; “series” is used to group related images by protocol (e.g., in magnetic resonance, it is common to find all the instances of a specific acquisition sequence in the same series); “instance” is the minor component in the DICOM data model. Usually, it consists of a single file containing metadata organized as DICOM tags and image data. In this work, we refer to DICOM instances as images.

Images from different modalities (mammography including tomosynthesis, magnetic resonance imaging [MR], and breast ultrasound) acquired from 2008 to 2021 were extracted from three regions of Sweden (Västmanland, Östergötland, and Södermanland) for the pilot study. The standard four-view mammogram examination is further explored and described. While the platform could be extended to process the MR and ultrasound modalities, these are stored for future use but due to the limited availability of commercial AI-CADx solutions for them, they are not processed. The images were stored in DICOM format. Before transfer to the virtual private cloud (VPC), they were pseudonymized at the institution using the Collective Minds (CM) Proxy (further described in Sec. 4.1) that locally receives an image and modifies the metadata before sending its pseudonymized version to our VPC storage service in a secure manner. Images for 105,181 examinations have been received so far from the three hospital regions.

### 3.2 Inferences

In the pilot study, three AI-CADe systems for mammography (Lunit INSIGHT MMG v1.1.7.2, Therapixel MammoScreen v2.1.0, and Vara v2.1) have been run in the cloud solution to produce the respective systems’ inferences. These were encrypted and transferred along with the images to the on-premises environment. The JavaScript Object Notation (JSON) file format was used to store Therapixel’s and Vara’s inferences, while for Lunit, the predictions were stored in a DICOM dataset. Generally, each file contained the inference results (the suspicion of presence of cancer) for a specific AI-CADe system for a particular study, i.e., a group of up to four images, generally with views Cranio-Caudal (CC) and Medio-Lateral Oblique (MLO) for each laterality. For a given examination, one or more inference rounds might occur depending on the images contained in the examination. For more details, we refer the reader to Appendix C for an explicit definition of when the instances are shown to the AI-CADe systems. A total of 175,403 inferences from the three vendors have been created for 35,575 studies (more than one inference per study is possible depending on the input case defined in Appendix C) that belong to 17,859 unique patients. The images that each model consumes from the images that are shown to them, depend on the system that will create the inference. The three systems currently installed accept the following input: Single-mammogram image (one by default), exam of four-mammogram images (all), exams from different time points (two), other modalities (none). The main case for the three algorithms is as described in Appendix C. Generally, four images are shown per inference. Although one of the algorithms outputs one score per image, the others output a score per laterality and it is in this way that the pilot of the platform stores the inferences. If more than two scores exist for a given examination, the maximum per laterality is used. For all of the algorithms, the output presents both segmentation of possible lesions as well as a classification score that tries to predict if cancer was present in the set of images, thus, including both CADe and CADx. In this work, we refer to both as inferences.

### 3.3 Assessments

The mammography screening assessments in Sweden are subject to double reading, where each examination is reviewed by two radiologists independently. If either of the radiologists flags the case as suspicious of breast cancer, a consensus discussion is held to determine whether the woman should be recalled for further diagnostic workup or not.^14^ In Sweden, the initial radiologist assessments and the decision in the consensus discussion are binary - positive or negative. In the health care systems of other countries, a graded system may be used to denote the level of cancer suspicion. The outcome of the individual assessments, as well as the final consensus decision, is recorded in the RIS of each region. For VAI-B, the RIS data extracted from each region were securely transferred to the on-premises storage and received as comma-separated values (CSV) tables with non-standard formats (Windows-1252 encoding, “$” as column delimiter, and “£” as string delimiter). Limited descriptions of the columns came with an attached text file. Each table row represents one decision of the double reading process used in Sweden. We obtained 1,146,786 final decisions for unique studies in the time spanning from January 01, 2008 to December 30, 2021.

The specific counts per combination of decisions (first, second, and final) and how these combinations were mapped to one of healthy, selection, technical recall, or N/A are shown in Appendix A, while the summary of the same data can be seen in Table 1.

**Table 1:** Summary of the extracted assessments. The status represents the collapsed final decision. It can be one of healthy (the radiologists did not find any indication of cancer), selection (the woman is recalled for further examination), or technical recall (an error in the acquisition procedure is detected by the radiologists). Missing information (N/A) is common since not all invited women participate in the study. The unique request number is the number of examinations assessed with the respective status.

Status	Unique request numbers in assessments	
Healthy	1,106,090	85.31%
Selection	39,320	3.03%
Technical recall	1376	0.11%
N/A	149,740	11.55%
**Total extracted**	1,296,526	100.00%

### 3.4 Cancer Outcomes

The AI-CADe inferences and the radiological assessments can be evaluated against a reference standard, which defines an examination as representing a cancer case or not. In VAI-B, the reference standard is determined by the *cancer diagnosis date* being within a particular follow-up time after the *screening date* of any given examination. Keeping the two mentioned dates in the database allows us to create alternative definitions of the reference standard. Since a cancer diagnosis close to the screening date (e.g., 3 months) was the result of screening radiologists’ work, a shorter follow-up time will bias the reference standard toward cases that were apparent to the radiologists. A longer follow-up time (e.g., 3 years) will decrease this bias but will increase uncertainty around whether the cancer was present in the breast when the mammogram was acquired.

The diagnosis dates were obtained from an extraction of the NKBC.^15^ The extraction resulted in multiple tables containing all the diagnosed cancer cases between 2008 and 2021 across the regions. NKBC provided a description of each column and the data type. Each row in the table represents one diagnosed cancer and is marked by a participant ID number, date of diagnosis, unique study ID, pathological, treatment, and patient characteristics. There were 12,345 unique patients with at least one cancer in the NKBC extraction.

## 4 Implementation

This section describes the implementation of the cloud and on-premises infrastructures. We also explain the data model used to create the database and the ingestion process for each data component.

### 4.1 Hybrid Architecture for Inference and Analysis

The platform is a hybrid solution consisting of an on-premises environment and a VPC for cloud services that satisfies the privacy and computational constraints. The latter supports the required infrastructure for the AI models from the vendors to run correctly while at the same time ensuring that the pseudonymized images are not accessible by the vendors.

**Fig. 2:**
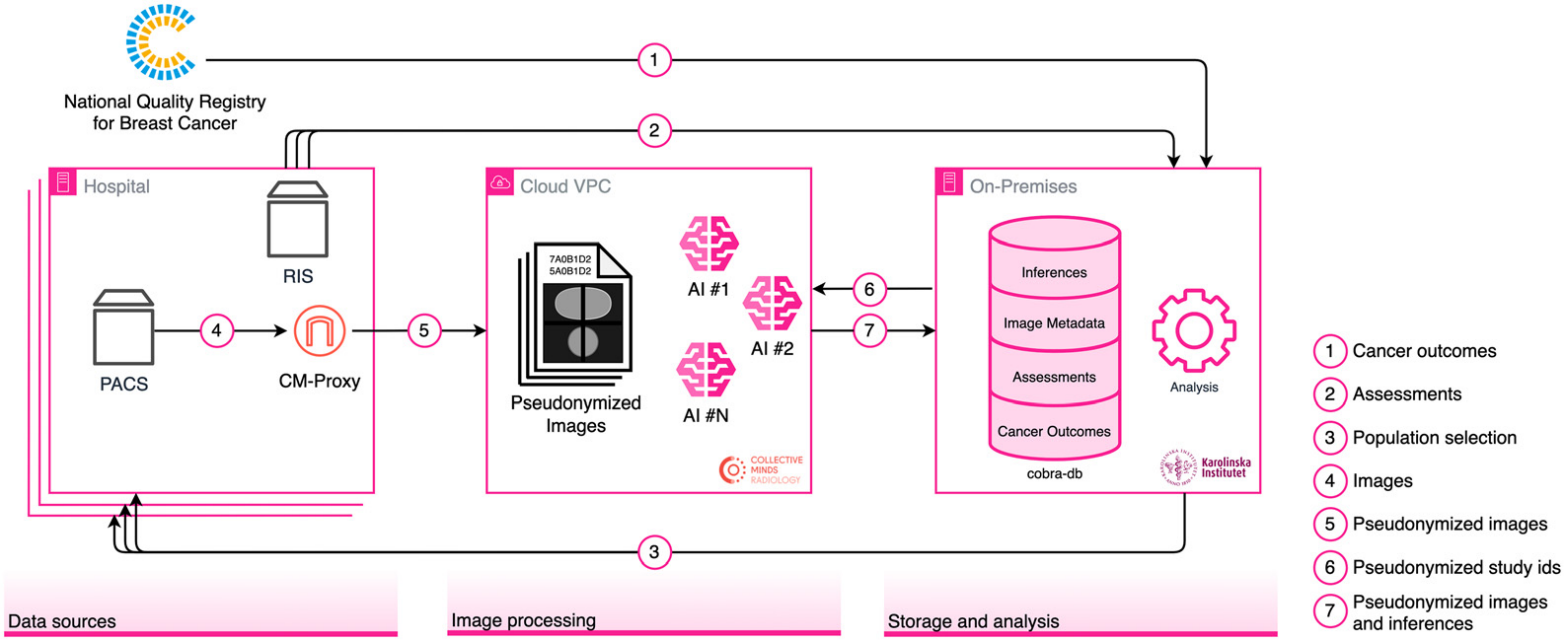
Overview of the order in which the data is transferred between the hospitals, NKBC, the VPC, and the on-premises environment. The arrows represent the path that the specified data follows.

**Fig. 3:**
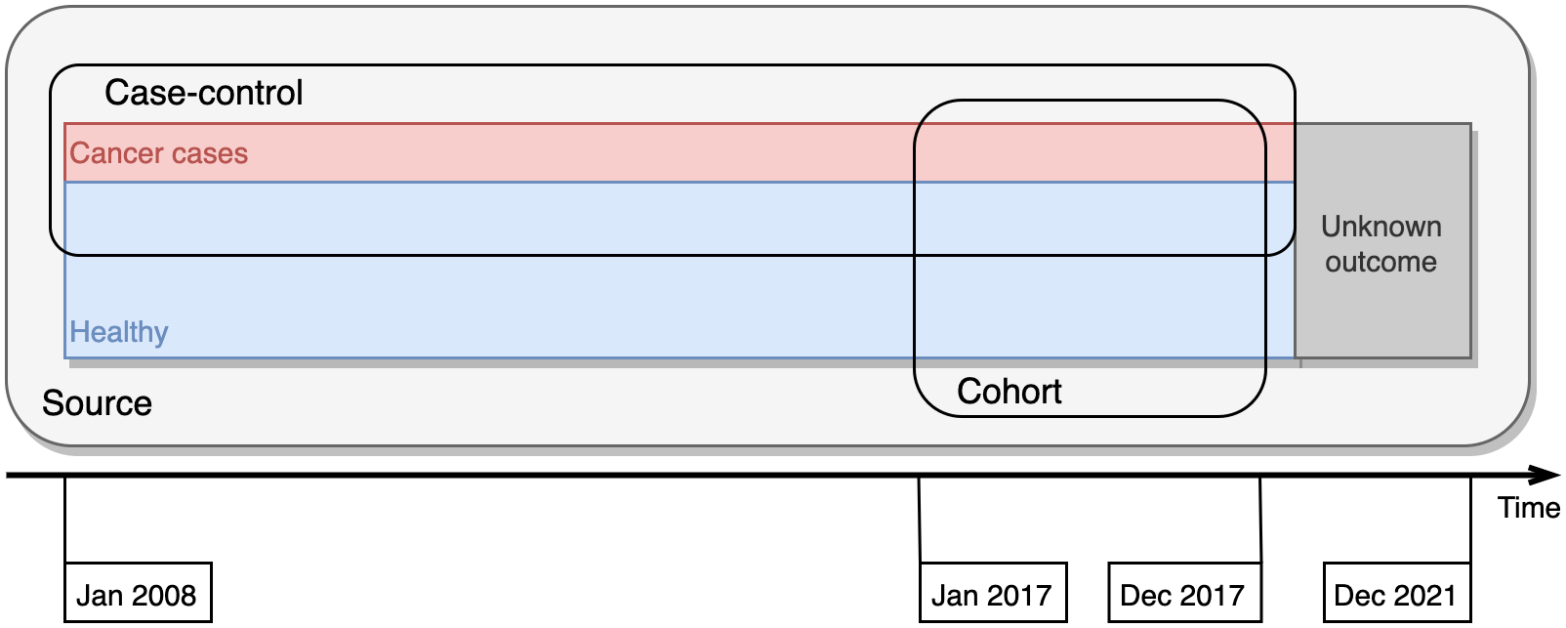
Conceptual overview of the different population selections “Source,” “Case-control,” and “Cohort” further explained in Table 2. Time is represented in the horizontal direction while the data from screening regarding healthy and cancer cases are shown in red and blue. The latest data has an unknown outcome if we consider the 36 months’ time that we use for the selection of the data.

The process of creating the COnsolidated BReast cancer Analysis-DataBase (COBRA-DB) requires several steps summarized as follows (arrows refer to Fig. 2):

I. *Identify suitable studies to be included*. In this step, the assessments and cancer outcomes were extracted from their respective sources (arrows 1 and 2) before being securely transferred to the on-premises environment for subsequent patient selection. The selection consisted of all patients that were diagnosed with cancer (all individuals in the cancer outcomes data) plus a randomized sample of controls (individuals not in the cancer outcomes data and with existing assessments data) at a 1:5 case-to-control ratio for each examination year, as well as a cohort-based population for all examinations of the year 2017. A list of the selected individuals was sent back to the hospitals (arrow 3). This list cannot be pseudonymized since the hospitals need the original identifiers to know what images to send. Simultaneously, both data components (assessments and cancer outcomes) were parsed, validated, pseudonymized, and stored in the database, further detailed in Sec. 4.3. A pseudonymized version of the selected individuals was sent to CM to assert that the expected data was received. The population selection is shown in Fig. 3 and the preliminary number of individuals selected for each population is shown in Table 2.
II. *Send images to the VPC*. The hospitals sent the images to the CM-Proxy (described in Appendix B) (arrow 4), which in turn uploaded the pseudonymized version of the images to the VPC (arrow 5). The specific pseudonymization procedure is described in Appendix B.
III. *Run the AI-CADe systems and obtain the inferences*. A selection of studies is requested (arrow 6) and their images are processed by the AI-CADe systems, previously installed in the VPC, to create inferences for each study.
IV. *Ingest data into the database*. The images and the inferences were sent to the on-premises server (arrow 7) to be ingested into the database.

**Table 2:** Population selection for each of the three regions. Participant refers to a woman that went to a screening examination; exam, to a screening examination.

	Population	Cancer cases		Healthy	
		Participants	Exams	Participants	Exams
Region	Source	(A) Diagnosed between 2008 and 2021	(B) All from participants in A	(C) Not diagnosed between 2008 and 2021	(D) All from participants in C
	Case-control	(E) With an examination 36 months before the diagnosis. All in F	(F) Within the 36 months before the diagnosis	(G) Random sample from C, approximately five times larger than E, and balanced for age and examination year	(H) All from participants in G
	Cohort	(I) All in J	(J) Acquired in 2017 and diagnosed within the next 36 months	(K) All in L	(L) Acquired in 2017 and not diagnosed within the next 36 months
Södermanland	Source	3108	10,755	79,808	335,842
	Case-control	2558	4244	11,762	12,780
	Cohort	330	330	9640	9640
Ostergötland	Source	3913	12,942	114,195	480,114
	Case-control	3311	5556	15,058	16,135
	Cohort	487	487	19,688	19,688
Västmanland	Source	2576	7953	68,646	286,326
	Case-control	2219	3487	9519	10,220
	Cohort	256	256	9129	9129

### 4.2 Image Processing in the VPC

This part of the implementation was performed by CM. Before the images were sent, a VPC in Amazon Web Services was prepared by installing the AI-CADe systems from the different companies with systems’ requirements specified by each vendor. The installation was done through manual configuration by the engineers from the companies and from CM. Then, the pseudonymized images were sent from the participating hospitals to the VPC, as explained in step II in Sec. 4.1. Upon the arrival of the images, an automatic process launched, which ingested the data into a structured repository of files, keeping images mapped to each pseudonymized individual. From there, the image processing pipeline started, which auto-scaled the infrastructure to parallelize over multiple servers hosting Docker containers that ran the inference and created results for the cohort and case-control populations. The populations were requested. We defined rules to input images to the AI-CADe systems to ensure comparability of the results, and the details can be found in Appendix C. For each study, the processing time fell in the range of 10-15 seconds per algorithm and given that the processing is done in a scalable way, multiple studies can be processed simultaneously. The images and inferences were synchronized to the on-premises environment for further analysis.

### 4.3 Database and Ingestion Pipeline

A MongoDB database was chosen to support the quality control and structuring of the extracted data^16^ due to the benefits described in the discussion section below.^17^ Scripts were developed to carefully ingest the extracted data into the database. The following design principles were incorporated into the data model^18^:

1. *Flat is better than nested*. Essential data lies directly at the root of document documents (akin to a JSON object) in the database. Infrequently accessed data is hierarchically embedded inside the same document.
2. *Sparse is better than dense*. As opposed to tables with missing values, in the database documents, the field does not exist if the value is missing.
3. *Readability counts*. The field names and values must have enough information to make their meaning intuitive. To improve readability, the snake case style is used to define the field names. Whenever possible, enumerations are defined with uppercased variables.

The designed data model and ingestion details are presented below in four parts that match the data components described in Sec. 3 Data. We use this style to indicate literal references to the python package or the database.

Each different document model (also known as schema) inherits the properties of the Entity type, which is the abstract base class for our data model. We exploited the similarity between the standard Dataclass in python and the MongoDB document-oriented data model to be able to map back and forth between database documents and python objects. Instances of a concrete Entity live in a collection with the same name as the Entity, e.g., all RadiologicalSeries instances are stored in the RadiologicalSeries collection of the database. Instances of an Entity contain a metadata field with information about the data model version, the date-time of creation, and the date-time of modification if any. The metadata also includes a project_name field in case documents from different projects with different ethical approvals are stored in the same database, and it allows us to filter the correct data depending on the user and application.

#### 4.3.1 Radiological data

Three of the entities in the database contain DICOM tags. These are the ImageMetadata, RadiologicalSeries, and RadiologicalStudy. They store the DICOM tags that are relevant to the instance. The most frequented information was parsed from the DICOM tags and stored at the document’s root. For example, in the RadiologicalStudy, the tags “Study Date” and “Study Time” are stored in the RadiologicalStudy.date attribute as an ISO date,^19^ allowing us to use the standard datetime python library or the MongoDB Query Language (MQL) aggregation $dateDiff to compare dates and making it straightforward to calculate time deltas when defining the study population.

We created an ImageMetadata document for each image file, which retains information about the original file’s name and location, the database-assigned ID for the series and study it belongs to, and the DICOM tags as human-readable text. Private tags were ignored. During the ingestion of the ImageMetadata collection, there were no assumptions about the folder structure or how the files were organized, as the root path was recursively searched for any file with the “.dcm” extension (DICOM file).

The database creation scripts were written to allow reusability in any context where a researcher wants to get the metadata of many DICOM files organized. A python package (also named cobra-db) was developed to standardize and facilitate frequent operations in the database (accessible at https://github.com/mammoai/cobra-db). The basic requirements are that the file-system with the images must be accessible from the computer creating the collection; the DICOM files must contain the SOPInstanceUID, the SeriesInstanceUID, and the StudyInstanceUID (or PatientID and StudyDate).

The script for ingesting images used multiprocessing to read many files on multiple drives simultaneously. For the images, the metadata is extracted using pydicom.^20^ Only the header of the file is read from the disk while the pixel data is ignored, reducing the time it takes to scan a drive.

For several years, our group has collected and studied the Cohort of Screen-Aged Women (CSAW),^14^ which contains data with the same components described above. We tested the needed processing time by running the ingestion script on all the CSAW data. For images that were, on average 25 MB, the system allowed us to read two images per second per worker for rotative hard disk drives (HDD) and 18 images per second for solid state drives (SSD). Using multiple processes, the drive limit was around ten images per second for HDD and above 90 images per second for SSD. Indexing 1M files from a single spinning disk took 23 hours. Furthermore, by running three disks in parallel (with 1,104,403, 1,024,146, and 220,882 images each), indexing the 2.3M images took 28 h. The ImageMetadata collection for indexing the 2.3M images of CSAW required 36GB of storage. For the VAI-B images received at Karolinska Institute until the time of submission (belonging to 105,181 examinations), the same script allowed us to ingest the metadata in 413 min.

With the ImageMetadata collection created, we aggregated images by any DICOM tag using MQL. We grouped by SeriesInstanceUID executing the following aggregation pipeline:

db.ImageMetadata.aggregate(

[{

$group: {

_id: '$dicom_tags.SeriesInstanceUID.Value',

image_ids: {$push: '$_id'}

}

}],

{allowDiskUse: true}

)

**Fig. 4:**
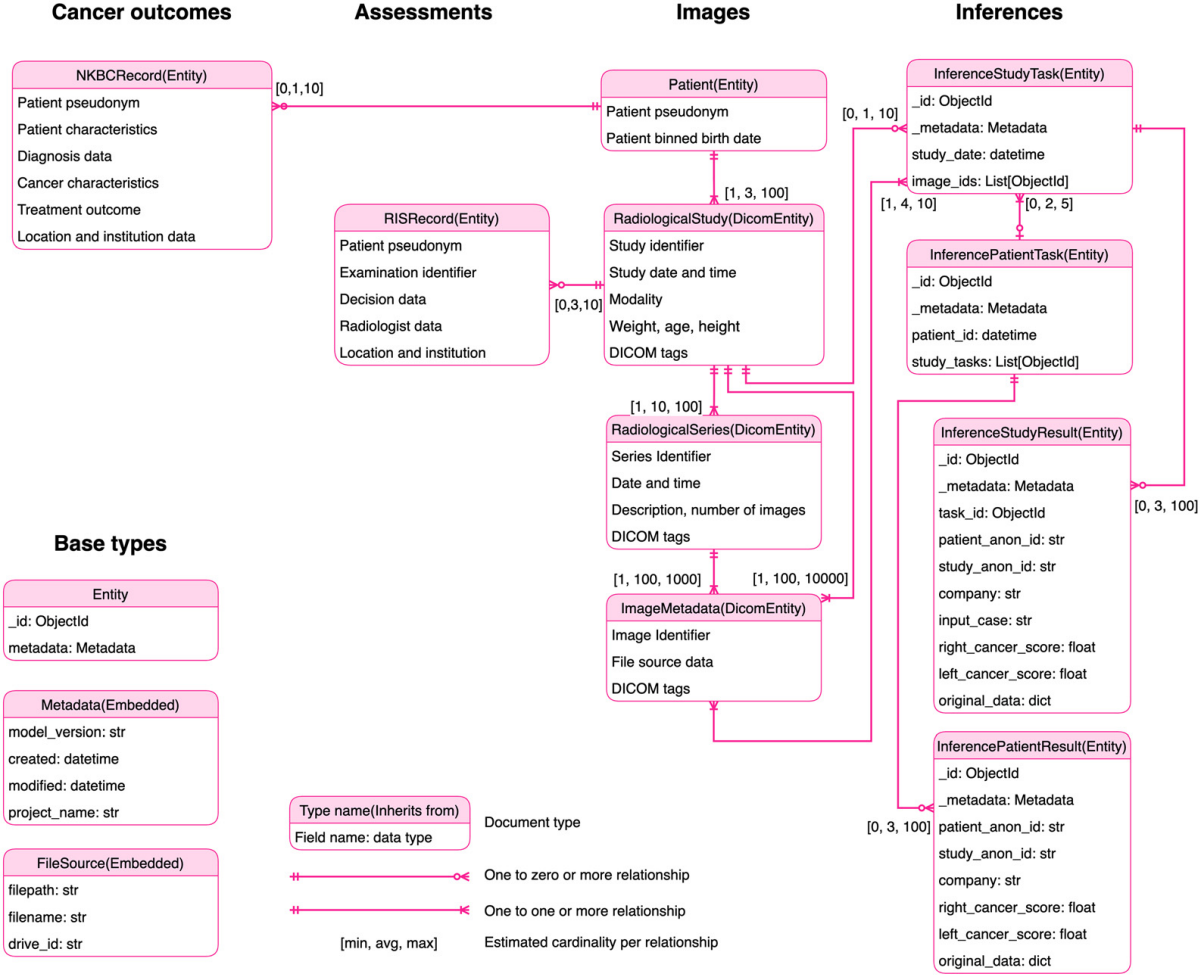
Entity relationship diagram describing the data model and specific content of the documents of the different collections of the database.

Images’ metadata was grouped into series, and the tags shared among all these images were passed to the RadiologicalSeries.dicom_tags dictionary. This operation considered three cases: (1) The tag had a value and was equal to all the other dictionaries. (2) The tag had a value that differed from at least one of the other dictionaries. (3) The tag was missing in some dictionaries but is equal in all others. For the first case, the field was kept. For the second case, there was a disagreement, and the field was discarded. For the third case, the majority decided if the field should be kept or not. We have called this aggregation method “intersection allow missing minority.” Once the shared DICOM tags dictionary was created, we continued to extract the essential information to the document’s root. The specific information that was extracted is shown in Fig. 4. Finally, the database-assigned ID of the series was set in each image document as series_id. Grouping the instances by the study was analogous to the one going from ImageMetadata to RadiologicalSeries. In this case, the images were grouped by the unique combinations of PatientID and StudyDate using the following query.

db.ImageMetadata.aggregate(

[{

$group: {

_id: {

patient_id: '$dicom_tags.PatientID.Value',

study_date: '$dicom_tags.StudyDate.Value'

},

image_ids: {$push: '$_id'}

}

}],

{allowDiskUse: true}

)

The DICOM tags of the images for the same patient and date were aggregated in the same way as described above (intersection allows missing minority). Then, the most useful information was parsed and stored in the root of the RadiologicalStudy document. A few fields like modality and study_uid were filled with the union of all the values seen for such study. This way, the researcher can, e.g., count all studies containing at least one image of mammography (MG) modality by executing the command db.RadiologicalStudy.countDocuments({modality:'MG'}). Finally, the studies were grouped by PatientID, and the Patient collection was populated. All the studies belonging to a unique patient were marked with the database-assigned ID of the patient.

#### 4.3.2 Cancer outcomes

The extracted data from NKBC were ingested into the database through a script that converted each row into a document of the type NKBCRecord. The data types specified in the documentation provided by NKBC were used to parse the received data. Before pseudonymization, the personal number (a 10- or 12-digit identification number of people residing in Sweden) was validated, as explained in Appendix D: Prehashing validation of Swedish personal number. Then, the personal number was hashed, and the other column names were converted to a readable version (e.g., the column “a_pat_sida” became field laterality, and the values “1” and “2” were converted to right and left, respectively). The columns were parsed according to the developed enumerations classes that served as documentation and validation of the data.

#### 4.3.3 Assessments

As for the cancer outcomes, the assessments were first converted to Unicode Transformation Format 8-bit (utf-8) text before being parsed as RISRecord documents and then stored in the database. The data were systematically validated against the supplied documentation to check that the variables in the provided tables contained valid values for all rows.

#### 4.3.4 Inferences

The inference data was transferred to the on-premises servers as the inferences were created from the VPC solution. In the definition of the InferenceStudyResult, we created one parser for each vendor’s JSON format. This way, we can extract the comparable metrics and store them in a shared data schema. The data from each inference was persisted in the original_data except for the encoded images that one of the companies’ outputs within the inference result.

## 5 Data Safety and Privacy

Ethical approval (EPM 2022-00186-01) was granted by the ethical review authority to perform the scientific study, including the extraction of the data and its usage for evaluation purposes. One of the critical challenges for the design and implementation of our platform was to manage the data safely and to create mechanisms to ensure the privacy of the individuals in compliance with GDPR while providing insightful results to the different stakeholders. In this section, we describe the adopted principles and actions. While creating the platform, we followed the nine principles of lawfulness, fairness, transparency, purpose limitation, data minimization, accuracy, storage limitation, integrity, and confidentiality.

The hybrid infrastructure provided the flexibility to minimize the data in two ways: (1) Only images and their inferences were stored in the cloud, while clinical details were pseudonymously stored in the on-premises database. (2) The number of transferred images was minimized by selecting them before their extraction from the hospitals. The image metadata that would not be needed was deleted before the images were transferred from the hospital to the VPC.

All information in the database was pseudonymized. Pseudonymization refers to the action of replacing the identity of a person with a consistent and fake identifier that cannot be reversed without a certain key.^21^ When extracting the images from the hospital, the fields that are required, but could compromise an individual’s identity, such as height, weight, and age, are rounded to coarser intervals to decrease the risk of re-identification further. The pseudonymization of images is described in Appendix B. After the data selection was made and the pseudonymized version of the assessments and cancer outcomes were ingested into the database, and the original data was locked in encrypted storage with strict access control.

At every stage of the process, the data is kept secure. At rest (in the cloud and on-premises infrastructures), it was stored in servers with physical hard-drive encryption, access control, and inside a VPN. For the assessments and cancer outcomes data, each hospital uploaded its data to a secured OneDrive location, where it was fetched by the researchers for further processing and pseudonymization. OneDrive has been designated by the IT department of the institute as safe for storing personal data since it has two-factor authentication and the access control can be granularly defined. The images were pseudonymized inside the hospital facilities and sent through an encrypted channel to CM. The commercial partner CM has been subject to an approval process before deciding to use their solution and then later audited to ensure that they conform with the agreed security protocols. Once the AI-CADe systems started processing the images, the vendors could no longer access the VPC.

## 6 Discussion

We have described the implementation of a hybrid platform for external validation of AI-CADe systems. There are clear advantages of processing the images in the cloud. While it is possible to build a GPU-enabled on-premises data center, using a cloud services provider requires fewer human resources, is faster, and is more scalable. Therefore, we created an intermediate cloud infrastructure that benefits hospitals, researchers, and AI-CADe vendors. The AI-CADe systems are not accessible to the researchers, and the images are not available to the vendors, protecting both the vendors’ intellectual property and the individuals’ personal data. The vendors can install and verify that their AI-CADe systems work correctly before the VPC is enclosed and the images are shown to the AI-CADe system.

One strength of the cloud part of our platform is the flexibility and scalability that the cloud infrastructure provides. This scalability shortened the estimated processing time for the inferences without increasing the computational costs, thanks to the possibility of doing massive cloud-based parallelization. In the pilot phase, the cloud and on-premises infrastructures were developed simultaneously in different organizations, requiring adapters to be implemented on-the-fly to interface them properly. Compared to the installation stage described in Sec. 4.2 a more standardized option is planned for future versions of our platform developed to handle automatic registration of new algorithms. This could be achieved by using containerization and defining the inputs that will be provided to the systems as well as the outputs that are expected. Further considerations are that the price of validation increments linearly to the number of exams processed, and that the system will only work in hospitals that allow CM to install the proxy to extract the images. Finally, since the images need to be sent to the VPC in Germany, it might be legally challenging for other countries, especially non-Europeans, to share their data with VAI-B.

In Sec. 4.1, we describe a series of steps that require interfaces that for the sake of the proof of concept, were bootstrapped to be manual interventions. In the future, these interfaces are planned to be standardized and automated as Application Programming Interfaces (APIs) with the benefits of lowering the probability of human error (and its propagation to further steps) and decreasing the time to validate an algorithm, which brings economic incentives to all stakeholders to participate in the external evaluation. More importantly, well-designed and automated interfaces are important since they can lower the risk of human error that could lead to data breaches.

Although there are multiple databases of breast cancer imaging^22^^–^^24^ and the usage of MongoDB for storing medical imaging has been previously explored,^25^^,^^26^ to the authors’ knowledge, there is no open-source database that considers the interactions between AI-CADe systems, radiologists, breast radiological imaging, and cancer outcomes data; and that at the same time provides the scripts for building a new instance of the database in a private server. Omi-db, for example, has also published a python package^22^ that allows for client connectivity to the already created OPTIMAM official database, unfortunately, it does not provide the database instance creation scripts, therefore it was not possible to use it with our private images in the proposed hybrid platform. The strengths of using a MongoDB database system for indexing the images are (1) *General purpose database.* As opposed to PACS which is strongly focused on medical imaging. Tabular and hierarchical data, such as DICOM metadata, can be stored. Thus, making the database adaptable to the needs of the research group. (2) *Indexing*. It allows indexing on any field or combination of multiple fields, which improves latency during custom queries. (3) *Expressive aggregation pipelines*. By using the MQL, it is possible to obtain data in the exact format and characteristics required for the downstream evaluation operations. (4) *Full ecosystem*. MongoDB provides the database engine and tools to manage and access the data. For example, Compass (the official graphical user interface) allows non-programmatic access to the database, while the different APIs provide access in all popular programming languages. On the limitations side, the database is currently focused only on metadata and text-based information. Although it holds the file paths for all images, it does not manage the files.

Carefully defining the data model as a python package also provides advantages. First, the data is ensured to have a level of quality. Second, the reusability of the data and the schema is enhanced, as other team members can use the API to connect to the database. With the open-sourced package, other engineers that work with medical imaging can build their database instance with their data and then adapt it or extend it to their needs. In the light of the rising interest in federated learning approaches,^27^ we consider that the cobra_db package could be used as a normalizing step in the process of creating the datasets at each node of a federated learning effort where each institution would first create their internal cobra_db instance that and then with standardized MQL language, export the desired dataset to be used with other federated learning and privacy-preserving libraries such as Syft.^28^

Altogether, VAI-B enables AI-CADe vendors to obtain validation results showing the accuracy and robustness of their system across patient age groups, mammography equipment types, cancer subtypes, and different hospitals. For participating hospitals, VAI-B allows them to obtain performance results for their specific clinical setting regarding patient population, radiologists, and mammography equipment. Judging from the engagement from the care providers and AI vendors thus far, the organizational and technical setup has been successful in establishing trust in the platform. The external evaluation of algorithms provided by VAIB is available to any hospital and company that wants to validate their algorithms. New hospitals are able to participate by sending their images and processing them with the algorithms that are already in the platform. This will provide them with performance results of algorithms on their data. To do this, the CMProxy should be installed inside the hospital and a selection of images must take place (arrows 2, 3, and 4 in Fig. 4). To obtain the ground truth to what the systems are compared, the cancer outcomes for each exam are required. For the current Swedish hospitals, our research group is who contacts the NKBC. On the other hand, new companies that would like to participate can send algorithms to receive feedback on the improvement opportunities. Therefore, the platform serves as a testing suite that allows the companies to benchmark and compare their own models and provide a quality control that is tuned to the specific image distribution of the targeted hospital. To do so, the companies must install their service on the platform. Such process currently takes place in a manual way but could be further automated.

## 7 Conclusion

We have designed and implemented the VAI-B platform—a multicenter platform for the external validation of AI systems in breast imaging. By creating a hybrid processing and storage solution, we addressed the privacy requirements of the different data providers while leveraging the scalability and flexibility of a cloud-based service. We have developed the method to extract, transform, and load (ETL) data in a database, which involved the development of the data model based on the available data. The conventions to access the database are written as an open-source python package that standardizes the frequent actions in the database. The same package allows the creation of a new database with any DICOM imaging data. We have also defined procedures for deidentification and for choosing the images consumed by the AI-CADe systems.

Most importantly, we have created a platform that will allow downstream evaluation of AI-CADe systems for breast cancer detection, which enables faster development cycles for participating vendors and safer AI adoption for the participating hospitals. The platform was designed to be scalable and ready to be expanded should a new vendor want to evaluate their system or should a new hospital wish to obtain an evaluation of different AI systems on their images.

## 8 Appendix A: Collapsing Assessments into a Single-Categorical Variable

For each screening examination, it was expected to have two independent decisions and one final decision. The possible choices were Discussion, Healthy, Selection, or Technical Recall. We must also consider the missing decision (N/A in Tables 2 and 3) as a possible choice. When aggregating the assessment decisions per examination, we have 39 permutations in the data. When making any kind of simplification (e.g., accuracy, sensitivity, or specificity scores), we need to create a single binary variable from the three decisions. The combinations of the first, second, and final assessments and how we collapsed them into one of the decision categories are shown in Table 3.

**Table 3:** Combinations of first, second, and final decisions that were present in the extracted assessments data.

Case	First decision	Second decision	Final decision	Unique request numbers	Count as
1	Healthy	Healthy	Healthy	812,089	Healthy
2	Healthy	N/A	Healthy	263,044	Healthy
3	N/A	N/A	N/A	149,740	N/A
4	Discussion	Discussion	Selection	17,986	Selection
5	Discussion	Healthy	Healthy	14,604	Healthy
6	Healthy	Discussion	Healthy	9796	Healthy
7	N/A	N/A	Selection	8967	Selection
8	Healthy	Discussion	Selection	5616	Selection
9	Discussion	Discussion	Healthy	5593	Healthy
10	Discussion	Healthy	Selection	3329	Selection
11	Discussion	N/A	Selection	3229	Selection
12	Discussion	N/A	Healthy	954	Healthy
13	Healthy	N/A	Technical recall	333	Technical recall
14	Discussion	Discussion	Technical recall	318	Technical recall
15	Discussion	Healthy	Technical recall	241	Technical recall
16	Healthy	Healthy	Technical recall	221	Technical recall
17	Healthy	Discussion	Technical recall	180	Technical recall
18	Healthy	N/A	Selection	132	Selection
19	N/A	N/A	Technical recall	74	Technical recall
20	N/A	Discussion	Selection	45	Selection
21	N/A	N/A	Healthy	9	Healthy
22	Discussion	N/A	Technical recall	8	Technical recall
23	N/A	Healthy	Selection	5	Selection
24	Selection	N/A	Selection	5	Selection
25	Healthy	Healthy	Selection	3	Selection
26	Selection	Selection	Selection	3	Selection
27	Technical recall	Healthy	Healthy	1	Healthy
28	Discussion	Technical recall	Technical recall	1	Technical recall

## 9 Appendix B: Collective Minds Proxy and Pseudonymization Procedure

The CMs Proxy is a lightweight server application installed locally in a hospital or institution. The proxy receives DICOM data sent directly from PACS or a modality, minimizes and pseudonymizes the header, and performs a secure (HTTPS) atomic transfer to a designated area in the CMs Radiology VPC. Therefore, ensuring that no identifiable information ever leaves the premise of a collaborating institution or hospital. For DICOM metadata, we use a modified version of the NEMA 2017c standard for de-identification. The following modifications were introduced to preserve data relevant to the tests we want to perform.

• Patient ID, Accession number, admission ID, interpretation ID. Performed procedure step ID, performing physician’s name, requested procedure ID, results ID, and study ID are hashed using “salted” SHA512/256 instead of blanked. Only the data controller has access to the salt.
• Retain patient characteristics option. The patient characteristics are binned to enhance privacy. Day is removed from day of birth (DOB), and only month and year are stored. Weight is rounded to intervals of 5 kg. Length is rounded to intervals of 5 cm. Age is rounded to intervals of 5 years.
• Acquisition time is preserved.
• Study description is preserved.
• Retain institution identity option is followed.
• Retain device identity option is followed.
• Retain longitudinal temporal information with full dates option is followed.

## 10 Appendix C: Input Selection Procedure

For comparability, it is imperative to control which specific images are given to each AI-CADe system. A set of tests are performed to classify and qualify each study.

• Assertion of headers,
  • Image laterality (R/L)
  • View position (MLO/CC)
  • Acquisition time
  • Instance number (fallback if acquisition time is not present)
• SOPInstanceClassUID present and prioritized as
  • 1.2.840.10008.5.1.4.1.1.1.2 - Digital Mammography X-Ray Image Storage - For Presentation
  • 1.2.840.10008.5.1.4.1.1.1.2.1 - Digital Mammography X-Ray Image Storage - For Presentation
  • 1.2.840.10008.5.1.4.1.1.1 - Computed Radiography Image Storage

The inference tasks are defined by the following logic with six different input cases. The selection targets to find instances from four different combinations of laterality and position (R-MLO, L-MLO, R-CC, L-CC).

• Input case 1 – One instance each of R/L-MLO and R/L-CC is found.
• Input case 2 – Instances for R/L-MLO and R/L-CC are found. Multiple instances exist for one or many.
  • Input case 2a – The most recent instances are selected.
  • Input case 2b – The oldest instances are selected.
• Input case 3 – One or many of R/L-MLO and R/L-CC are missing. Only one instance of each existing combination is found.
• Input case 4 - One or many of R/L-MLO and R/L-CC are missing. Multiple instances exist for one or many combinations found.
  • Input case 4a – The most recent instances are selected.
  • Input case 4b – The oldest instances are selected.

Please note that input cases 1 and 2a/b will generate input sets with four instances, while input cases 3 and 4a/b will have less than four.

## 11 Appendix D: Prehashing Validation of Swedish Personal Number

Various formats exist to represent the same personal number (e.g., 19000101-5678, 190001015678, and 01015678), resulting in different hashes. The personal numbers were validated with the following actions. First, all nonalphanumerical characters were deleted, then the remaining characters were checked for the following characteristics:

• All characters were alphanumerical: worth one point.
• Correct length (12 characters YYYYMMDDXXXX): worth two points.
• A valid date could be parsed from YYMMDD: worth four points.
• The last digit was a correct checksum: worth eight points.

The points are summed, creating a score from 0 to 15, interpreted as a binary flag of 4 bits. The score is stored along the hashed Personal Number and helps assert comparability of two hashed personal numbers.

## Code, Data, and Materials Availability

Code for the VPC implementation is available on request to Collective Minds

Code for each AI-CADe system is available upon direct agreement with the vendors.

Code for creating the database is openly available at https://github.com/mammoai/cobra-db.
